# Effectiveness of Distance Technology in Promoting Physical Activity in Cardiovascular Disease Rehabilitation: Cluster Randomized Controlled Trial, A Pilot Study

**DOI:** 10.2196/20299

**Published:** 2021-06-18

**Authors:** Sanna Hakala, Heikki Kivistö, Teemu Paajanen, Annaliisa Kankainen, Marjo-Riitta Anttila, Ari Heinonen, Tuulikki Sjögren

**Affiliations:** 1 University of Jyväskylä Jyväskylä Finland

**Keywords:** cardiac rehabilitation, rehabilitation, cardiovascular diseases, technology, exercise, randomized controlled trial, clinical trial

## Abstract

**Background:**

Physical activity is beneficial for cardiovascular rehabilitation. Digitalization suggests using technology in the promotion of physical activity and lifestyle changes. The effectiveness of distance technology interventions has previously been found to be similar to that of conventional treatment, but the added value of the technology has not been frequently studied.

**Objective:**

The aim of this pilot study was to investigate whether additional distance technology intervention is more effective in promoting physical activity than non-technology–based treatment in 12 months of cardiac rehabilitation.

**Methods:**

The cardiovascular disease rehabilitation intervention consisted of three 5-day inpatient periods in a rehabilitation center and two 6-month self-exercise periods at home in between. Participants were recruited from among cardiac patients who attended the rehabilitation program and were cluster-randomized into unblinded groups: conventional rehabilitation control clusters (n=3) and similar rehabilitation with additional distance technology experimental group clusters (n=3). Experimental groups used Fitbit Charge HR for self-monitoring, and they set goals and reported their activity using Movendos mCoach, through which they received monthly automated and in-person feedback. Physical activity outcomes for all participants were measured using the Fitbit Zip accelerometer and the International Physical Activity Questionnaire.

**Results:**

During the first 6 months, the experimental group (n=29) engaged in light physical activity more often than the control group (n=30; mean difference [MD] 324.2 minutes per week, 95% CI 77.4 to 571.0; *P*=.01). There were no group differences in the duration of moderate to vigorous physical activity (MD 12.6 minutes per week, 95% CI –90.5 to 115.7; *P*=.82) or steps per day (MD 1084.0, 95% CI –585.0 to 2752.9; *P*=.20). During the following 6 months, no differences between the groups were observed in light physical activity (MD –87.9 minutes per week, 95% CI –379.2 to 203.3; *P*=.54), moderate to vigorous physical activity (MD 70.9 minutes per week, 95% CI –75.7 to 217.6; *P*=.33), or steps per day (MD 867.1, 95% CI –2099.6 to 3833.9; *P*=.55).

**Conclusions:**

The use of additional distance technology increased the duration of light physical activity at the beginning of cardiac rehabilitation (for the first 6 months), but statistically significant differences were not observed between the two groups for moderate or vigorous physical activity or steps per day for both 6-month self-exercise periods.

**Trial Registration:**

ISRCTN Registry ISRCTN61225589; https://doi.org/10.1186/ISRCTN61225589

## Introduction

Cardiovascular rehabilitation (CR) aims to reduce cardiovascular risks, encourage healthy behaviors and adherence, reduce disability, and promote an active lifestyle. Cardiovascular disease (CVD) care is multidimensional, including nutritional counseling, risk factor management, psychosocial interventions, and the promotion of physical activity [1]. Physical activity benefits patients with CVD by reducing the risk for further cardiac events [2], and exercise-based CR has been proven to reduce overall and cardiovascular mortality [3]. In addition, light-intensity physical activity has been shown to be beneficially associated with obesity, markers of lipid and glucose metabolism and mortality [4]. However, the physical activity engagement of cardiac patients has been below the threshold with respect to gaining improvements in cardiorespiratory fitness [2].

Home-based CVD rehabilitation has been found to be as effective as center-based rehabilitation in improving clinical and health-related quality of life outcomes [5]. Digitalization has given rise to the application of distance technology toward physical activity-promoting rehabilitation [6]. Technology-based physical activity-promoting distance interventions have been shown to be as effective as comparable interventions delivered conventionally (ie, paper-based materials or in-person meetings) [7]. Compared to usual care, technology-based distance interventions are more effective in increasing physical activity, especially among patients with medical diagnoses [8-10]. Our preliminary systematic reviews showed that technology may be a promising tool for promoting physical activity in CR [8,9]. However, there is insufficient evidence on the additional value of distance technology in interventions promoting physical activity for patients with CVD.

This pilot study aimed to investigate, at the individual participant level, whether internet software and activity monitoring in addition to a 12-month conventional cardiovascular distance rehabilitation effectively promotes light or moderate to vigorous physical activity (MVPA) or an increase in steps per day, compared to similar rehabilitation regimes without distance technology. Cluster randomization was chosen to control the potential cross-contamination between experimental and control groups, since the intervention took place in standard cardiac rehabilitation group-based courses. The rehabilitation program consisted of two 6-month home-based rehabilitation periods in between three 5-day inpatient courses.

## Methods

### Design

This cluster randomized controlled trial pilot study (ISRCTN61225589) assessing the effect of additional distance technology-based rehabilitation on patients with CVD was conducted between September 21, 2015, and November 30, 2017. The study protocol was approved by the Ethics Committee of the Central Finland Health Care District. The 12-month CR program was executed in groups of 10 rehabilitees each, which is standard practice at the Peurunka rehabilitation center. Sample size was defined by the rehabilitation groups that began the rehabilitation during the years 2015-2016. Physical activity outcomes were measured 3 times during the intervention: at baseline, 6 months, and 12 months. Participants or caregivers were not blinded to the intervention. However, the physical activity outcome assessor (author SH) and the researchers (authors TP and AK) who performed the statistical analyses of the results were blinded to the treatment allocations of the groups.

### Participants and Randomization

Participants were recruited from among patients with CVD who attended the CR program at a rehabilitation center in Finland. The eligibility criteria of the participants included age (18 years or older), diagnosed cardiovascular risk factors, angina pectoris with physical working capacity limitations, myocardial infarction, coronary artery bypass graft surgery, or coronary angioplasty. Exclusion criteria included musculoskeletal disorders, cognitive or memory impairment, or if the independent use of computer or remote technology application was not possible.

Participants had been allocated into 6 groups by officers of Social Insurance Institution of Finland, who also scheduled the inpatient periods for each group. Officers in charge of the allocation were not informed about the research project. Randomization was designed by three members of the research group (authors HK, TS and AK), and it was completed before interventions or any of the rehabilitation courses started by two members of the research group (authors HK and TS) and one person outside the research group generating the random allocation sequence.

Participants were cluster randomized in cluster pairs (1 and 2, 3 and 4, 5 and 6) by picking up numbered papers (either “A” or “B”) that issued to which treatment allocation the group belonged. Pairwise randomization of clusters bypassed any systematic effect bias due to the season in which the rehabilitation was conducted. The groups of rehabilitees were randomized to control and experimental clusters, one of each starting in autumn, winter, and spring. After randomization, rehabilitation center staff individually enrolled participants who provided written consent in the intervention. Participants were assigned to the interventions by two members of the research group (authors HK and TS).

### Conventional Cardiac Rehabilitation

Cardiac rehabilitation courses were arranged by the Social Insurance Institution of Finland, and in this study, the courses were held in and the data were collected from one rehabilitation center. The group-based courses were driven by a multidisciplinary team and consisted of meetings with a doctor; physiotherapist; nurse; and, optionally, a social worker, psychologist, or dietitian. The CR courses aimed to promote multidimensional self-efficacy by focusing on psychosocial factors related to coping with CVD in daily life. During the course, participants obtained information about CVD, counseling for managing daily activities with heart illness, and group discussions with peers, in addition to physiotherapy and aerobic exercise. During the inpatient periods in the rehabilitation center, participants performed health- and functioning-related tests.

A 12-month rehabilitation course, that both groups attended, consisted of three 5-day inpatient face-to-face rehabilitation periods in the rehabilitation center (at the beginning of the study, at month 6, and at month 12). The rehabilitation program promoted adaptation to life with CVD. Promoting physical activity was one part of the content, which aimed to reduce the barriers CVD rehabilitees have with respect to exercising [11]. Participants were provided information on the health benefits of physical activity based on the American Heart Association (AHA) recommendations [12] and encouraged to exercise in accordance with their condition. The walking goal was 10,000 steps per day. Individual goal setting of total physical activity followed the Specific, Measurable, Achievable, Relevant and Time-bound goal setting framework [13]. Between inpatient periods, participants followed their designated exercise programs. Participants in the conventional cardiac rehabilitation control group received the usual rehabilitation program, which was identical to the experimental group but did not contain technology. In the control group, the instructions on how to perform exercises, goal setting, and self-monitoring were paper-based for the control group. Goals were determined using the Goal Attainment Scale [14].

### Additional Distance Technology in Conventional Cardiac Rehabilitation

Participants in the additional distance technology experimental group received an additional distance technology program, which aimed to promote health and functioning-related lifestyle changes, such as physical activity and healthy nutrition. Participants set and monitored their individual goals and received instructions on how to perform exercises via the Movendos mCoach internet software [15]. The experimental group was instructed and motivated to perform physical activity self-monitoring with a wrist-worn Fitbit Charge HR [16] activity monitor. The Fitbit Charge HR displayed steps per day, energy expenditure, heart rate, and the quantity and quality of sleep. At the beginning of the intervention, the experimental group received 1.5-hours face-to-face support on the use of activity monitoring technologies from an information technology specialist, a nurse, and a psychologist. During the second inpatient period, 6 months later, the participants attended another 30-minute counselling session. In addition, the participants received a tutorial video on how to use Fitbit Charge HR and Movendos mCoach. The participants were contacted twice a month through the application. They received one automatic prompt to engage in physical activity, and the second contact was made by a physiotherapist who gave feedback of the activity recorded in the application.

### Measures

Objective physical activity for all participants, in terms of steps per day, light physical activity (LPA), and MVPA were measured individually from each participant using a hip-worn Fitbit Zip (San Francisco, CA) accelerometer during the outpatient period for one week at 0, 6, and 12 months. Participants were instructed to use the device all the time while awake and not during sleep. The Fitbit Zip device automatically defines thresholds for the outcomes between LPA and MVPA. The days participants walked 1000 to 30,000 steps per day were included in the analysis. Subjective MVPA was measured using the International Physical Activity Questionnaire (IPAQ) [17], in which the participants recalled their activity over the last 7 days. During the inpatient periods, participant clusters were asked about the possible harms and other problems related to the use of technology and the intervention. Subjective adherence to the treatment was measured individually with a separate questionnaire (S. Hakala, unpublished data, October 2020). Adherence on using internet software was measured by analyzing the number of recordings made to the software, and the number of messages sent to the care provider during the 12-month intervention.

### Statistical Analyses

Two researchers (authors AK and TP) conduced the statistical analyses using IBM SPSS Statistics for Windows, Version 25.0 [18]. Outcome analysis was blinded, analyzing outcomes from group A and group B. After all the analyses were made, the encrypted treatment allocations of both groups were revealed. Absolute scores of outcomes measured with Fitbit Zip and IPAQ were used in analysis of changes in outcomes from baseline to 6 months, and from 6 to 12 months. Linear mixed models evaluated the effectiveness of the intervention on physical activity outcome. An independent samples *t* test (two-tailed) or Mann-Whitney *U* test analyzed differences between groups. Mean difference (MD) values described the differences between groups’ means, whereas positive values favored the experimental group and negative values favored the control group. In-group changes were analyzed based on tests of normality and using a paired samples *t* test (two-tailed) or Wilcoxon signed-rank test. Confidence intervals were reported with point estimates.

## Results

### Participants

**Figure 1:**
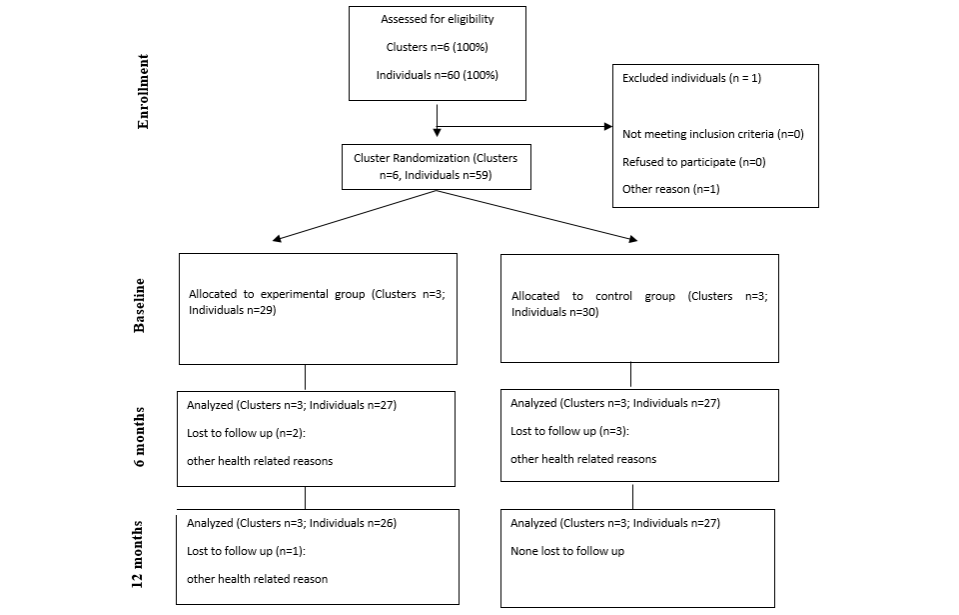
Flowchart of the cardiac rehabilitation intervention. CONSORT: Consolidated Standards of Reporting Trials.

The participants’ mean age was 60 (SD 6.0; range 41-66) years, and 81% (48/59) of the participants were male (Table. 1). The level of physical activity in both groups was between low and moderate at baseline (see Multimedia Appendix 1). Bypass surgery was performed for 9 (15%) out of 59 participants, and angioplasty was performed for 75% (44/59) of participants either once (36/44, 61%), twice (5/44, 8%) or more (3/44, 5%). Cardiovascular operations were performed 3 to 6 months before the intervention (10/50, 20%), 6 to 12 months before the intervention (24/50, 48%), more than 12 months before the intervention (14/50, 28%), or during the intervention (2/50, 4%). The dropout rate during the intervention was 10% (6/59; Figure 1).

**Table 1:** Baseline characteristics in the conventional cardiac rehabilitation control group and the additional distance technology intervention experimental group.

Baseline characteristics	Control group (n=30) mean (SD)	Experimental group (n=29) mean (SD)	*P* value
Age (years), mean (SD)	59.2 (6.1)	59.7 (6.0)	.73
Female, n (%)	4 (13)	7 (24)	.36
BMI, mean (SD)	28.5 (4.3)	29.0 (5.2)	.69
6 minutes walking test (meters), mean (SD)	661.5 (83.7)	610.0 (68.1)	.40
Steps per day, mean (SD)	7269 (2565)	6614 (3859)	.48
LPA^a^ (min/week), mean (SD)	177 (55)	150 (58)	.10
MVPA^b^ (min/week) , mean (SD)	176 (130)	184 (165)	.85
Uses a computer, n (%)	23 (77)	26 (90)	.28
Uses a smartphone, n (%)	22 (73)	22 (76)	.87
Owns self-monitoring devices for PA^c^, n (%)	11 (37)	13 (45)	.58

^a^LPA: light intensity physical activity.

^b^MVPA: moderate to vigorous physical activity.

^c^PA: physical activity.

### Physical Activity Changes From 0 to 6 Months

**Figure 2:**
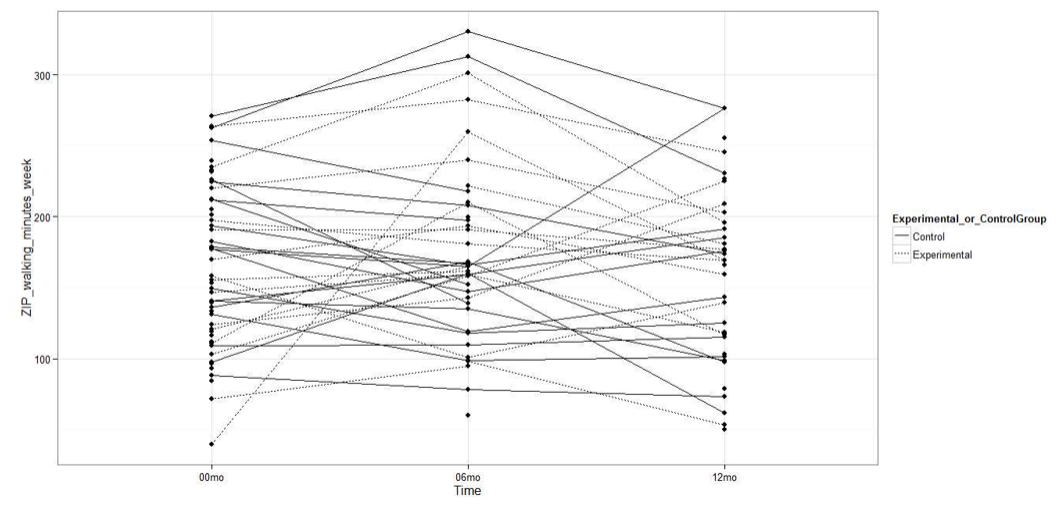
Light physical activity measured with the accelerometer at baseline, 6 months, and 12 months.

**Figure 3:**
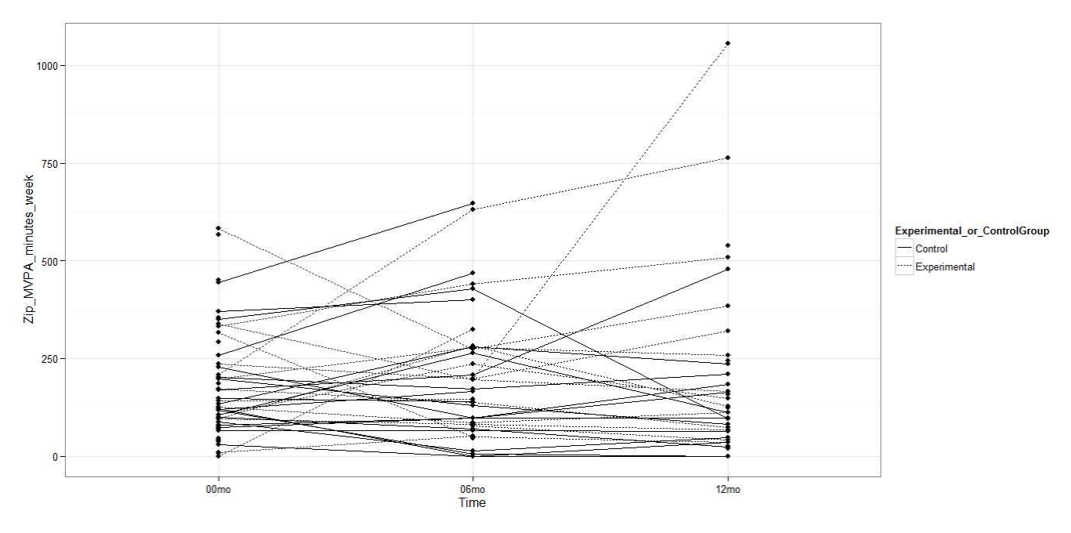
Moderate to vigorous physical activity measured with the accelerometer at baseline, 6 months, and 12 months.

**Figure 4:**
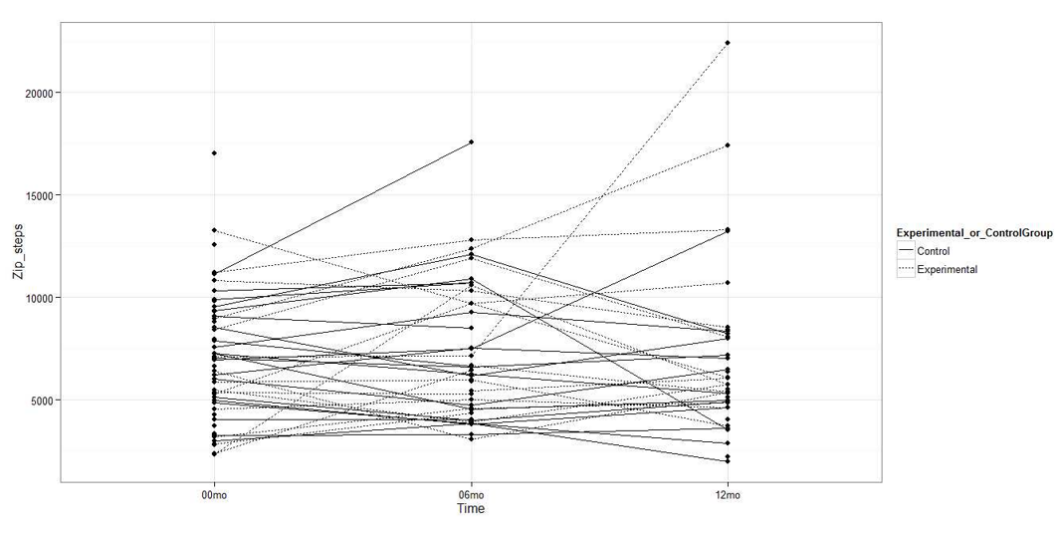
Steps per day measured with the accelerometer at baseline, 6 months, and 12 months.

The experimental group had a greater increase in the minutes per week of LPA measured with the Fitbit Zip accelerometer compared to the control group (MD 324.2 min, 95% CI 77.4 to 571.0; *P=*.01; Figure 2), but there were no statistically significant differences between groups in the minutes per week of MVPA (MD 12.6 min, 95% CI –90.5 to 115.7; *P=*.82) or in steps per day (MD 1084.0 steps, 95% CI –585.0 to 2752.9; *P*=.20; Figures 3 and 4). Likewise, there were no statistically significant differences between groups with respect to the duration of MVPA measured by the IPAQ (MD 34.0 min, 95% CI –327.3 to 395.3; *P=*.85).

### Physical Activity Changes From 6 to 12 Months

No statistically significant differences were observed between the experimental and control groups in the accelerometer-measured LPA minutes per week (MD –87.9 min, 95% CI –379.2 to 203.3; *P=*.54; Figure 2), MVPA minutes per week (MD 70.9 min, 95% CI –75.7 to 217.6; *P=*.33), or steps per day (MD 867.1 steps, 95% CI –2099.6 to 3833.9; *P=*.55; Figures 3 and 4). As such, there were no differences between groups in the MVPA minutes per week measured with the IPAQ (MD –292.2 min, 95% CI –755.6 to 171.2; *P=*.21).

### Adherence and Side Effects of the Treatment

During the 12-month intervention, participants in the experimental group made an average of 98 (SD 167; range 0-716) recordings regarding their physical activity in Movendos mCoach and sent an average of 6.4 (SD 6; range 0-26) messages to the care provider. The results from a subjective questionnaire indicated that on a scale from 1 to 7, in which 1 indicated “totally agree” and 7 indicated “totally disagree,” the participants almost or somewhat agreed they had actively used the Fitbit Charge HR (mean 2.4, SD 2.0). Participants somewhat disagreed that they had actively used Movendos mCoach (mean 4.9, SD 2.2). In total, 14% (4/29) of participants reported developing eczema as a result of the wristband of the Fitbit Charge HR. No other harms were reported.

## Discussion

In this 12-month cluster randomized trial, additional distance technology-based intervention effectively increased LPA during the first 6-month period of cardiac rehabilitation and maintained the achieved level of physical activity from 6 to 12 months. The novelty of this pilot study was the significant finding that distance technology brings added value in increasing LPA compared to similar rehabilitation interventions without technology. Previous studies have shown that distance technology-based physical activity interventions are approximately as effective as comparable conventional care in increasing physical activity [7], which is in line with this study, as no differences between groups were observed in MVPA or steps per day.

Recommendations on physical activity for special populations differ from those addressed to healthy people [19]. Adherence to physical activity recommendations has been low in both males and females with a history of cardiac events and previous studies adduced the difficulties in prescribing appropriate exercise regimens and achieving patient adherence in CVD rehabilitation [20]. Patients with CVD must overcome various barriers to exercise, such as having minor injuries, lack of time, or fatigue [11]. Although they would benefit from high-frequency training, improving their maximum aerobic capacity and muscle function, the fear of exercising is highly disruptive for 20% of cardiac patients [21]. The AHA recommends performing LPA given its recently studied health impacts [12]. LPA has been found to be favorable for multiple health-related outcomes, such as BMI, waist circumference, C-reactive protein, insulin resistance [4] and blood plasma glucose [22]. Obtaining a sufficient level of LPA is important, especially in the subacute phase of CVD rehabilitation, providing a basis for more intensive and independent exercising during the next phase of the rehabilitation (ie, intensive outpatient therapy). Our results illustrating substantial increases in LPA of 324 minutes per week in the experimental versus control group during the first 6 months are also clinically valuable.

We chose to use cluster randomization for practical and ethical reasons. In cluster randomization all the rehabilitees from the same group are enrolled to the same treatment allocation. As the intervention was executed in groups of participants, following the standard practice in cardiac inpatient rehabilitation, cluster randomization controlled the cross-contamination of experimental and control groups. Pairwise randomization of clusters matched for the possible seasonal influences to be similar in both groups. This was reasonable, as the weather conditions vary between seasons in Finland. Since no differences between the groups at baseline were observed in most prognostic factors related to the results, randomization was shown to be successful.

This study used Fitbit Zip to measure physical activity, as it was considered suitable for research purposes at the time of planning the intervention. It is reasonable to assume the rapid acceleration of technology brought forth more accurate devices for measuring physical activity, which provides a basis for evaluating previously used versus newly developed technologies, including their accuracy, as more studies are conducted. Problems with the accuracy of Fitbit Zip occur mainly with more intensive activities, which subacute phase cardiac rehabilitees are not normally engaging in, and with resting time, which was not investigated in this study. During normal walking, Fitbit Zip records measurements with greater accuracy when placed in the torso region [23]. In this study, Fitbit Zip was placed on the hip; although wrist-worn accelerometers have been found to be more comfortable to wear [24], the long durations of the self-exercising periods require devices with greater memory capacities, which hip-worn accelerometers have. Physical activity was measured with both the objective accelerometer and subjective questionnaire. However, objective physical activity could have been measured with periods longer than 1 week to avoid the loss of data.

The strength of this study is the comparative design of both experimental and control interventions. The distance technology intervention was also well-tolerated by the participants, which may have resulted from intensive counselling on the use of technology and available practical support during the intervention. The intervention did not cause side effects or harm participants. The rehabilitation center is well-experienced in managing cardiac rehabilitation programs, and the intervention was designed with a multi-professional and interdisciplinary research group.

The limitations of this study relate to the sample size, which could have been larger. Nevertheless, we were able to focus on improving the adherence to the intervention with in-person counselling sessions at the beginning and in the middle of the intervention. For instance, to ensure the correct use of the Fitbit HR Charge and Movendos mCoach, a tutorial video was made for the participants in the experimental group. Clear instructions on how to use technology have been found to be important among older people [25]. Despite the tutorial video and the in-person consultations, the participants faced multiple technical challenges or sometimes forgot to use the device. In the future, the use of technology could improve with better compatibility between the activity monitor and computer or the implementation of less expensive, more advanced smartphones. In addition, future studies should investigate whether a more intensive use of technology could promote physical activity at different intensity levels and for longer periods of time. Likewise, the diversity of the participants pertaining to technology use and the ability to adapt to new technologies should be taken into account at the individual level [26]. Qualitative research is essential to further explain findings from CVD-related physical activity studies, develop effective physical activity motivation and encouragement methods, and thereby enhance patient management. Special focus should be given to increasing MVPA by improving the types of physical activity intervention.

In conclusion, our findings indicate additional distance technology in cardiac rehabilitation may be effective in promoting LPA; however, technology provided no added value compared to conventional rehabilitation alone in MVPA or in steps per day. In the future, more studies are needed first to determine effective methods for promoting physical activity among different populations and, then to resolve which types of technology would best serve this purpose. However, the rapid acceleration of technology development requires significant investments in the research field.

## Appendix

Multimedia Appendix 1 Physical activity changes during the 12 months of cardiac rehabilitation.

Multimedia Appendix 2 CONSORT EHEALTH-checklist (V 1.6.1).

## Abbreviations

AHA: American Heart Association
CR: cardiovascular rehabilitation
CVD: cardiovascular disease
IPAQ: International Physical Activity Questionnaire
LPA: light physical activity
MD: mean difference
MVPA: moderate to vigorous physical activity
