# Supplementary figures and images for: Effectiveness of Distance Technology in Promoting Physical Activity in Cardiovascular Disease Rehabilitation: Cluster Randomized Controlled Trial, A Pilot Study
Source: JMIR Rehabil Assist Technol. 2021 Jun 18;8(2):e20299. doi: 10.2196/20299 (PMC8277324; doi:10.2196/20299)

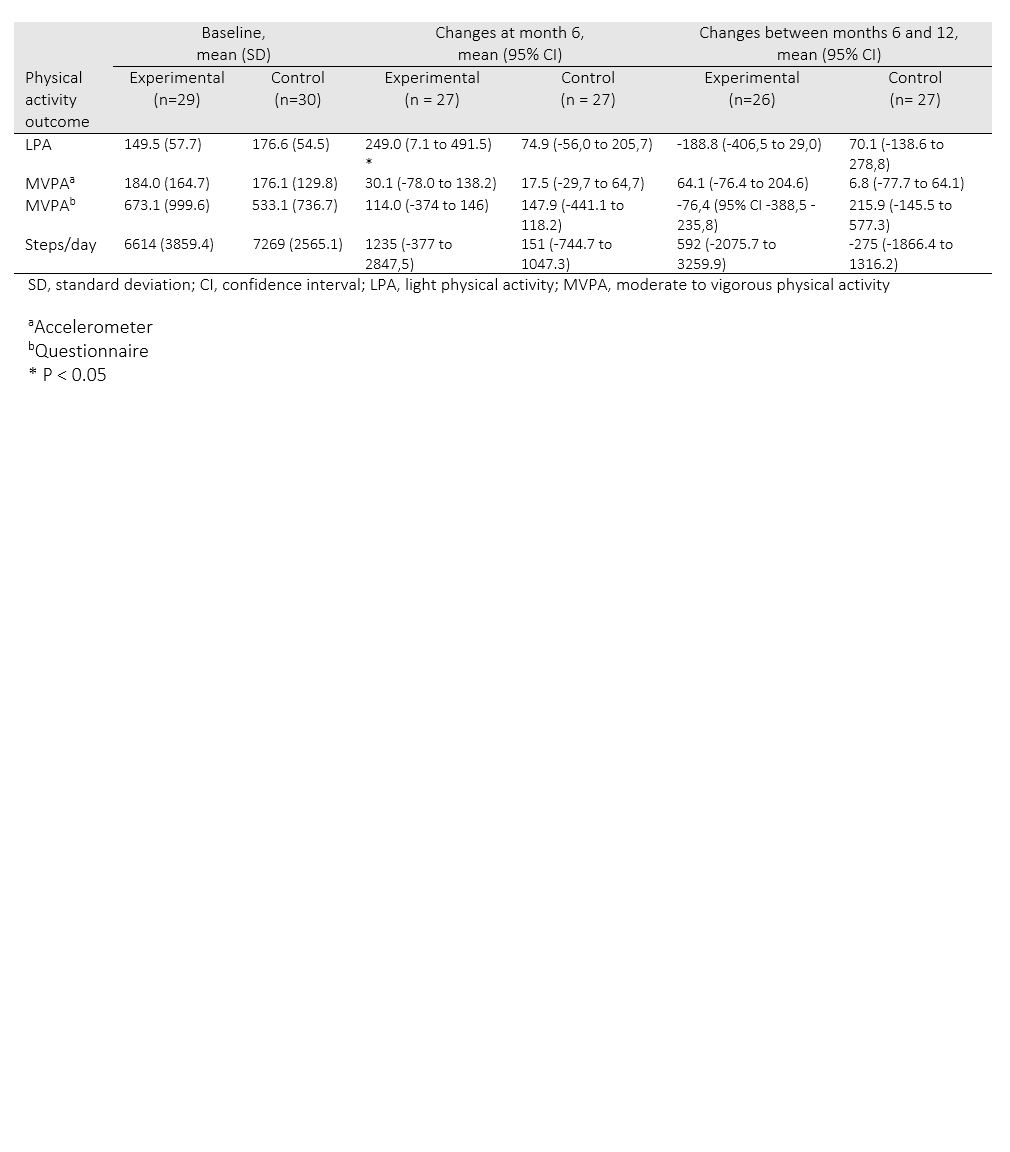

Supplement: Multimedia Appendix 1 [file rehab_v8i2e20299_app1.png]
